# Cross Entropy of Neural Language Models at Infinity—A New Bound of the Entropy Rate

**DOI:** 10.3390/e20110839

**Published:** 2018-11-02

**Authors:** Shuntaro Takahashi, Kumiko Tanaka-Ishii

**Affiliations:** 1 Graduate School of Frontier Sciences, The University of Tokyo, Chiba 277-8561, Japan; 2 Research Center for Advanced Science and Technology, The University of Tokyo, Tokyo 153-0041, Japan

**Keywords:** entropy rate, natural language, language model, neural language model

## Abstract

Neural language models have drawn a lot of attention for their strong ability to predict natural language text. In this paper, we estimate the entropy rate of natural language with state-of-the-art neural language models. To obtain the estimate, we consider the cross entropy, a measure of the prediction accuracy of neural language models, under the theoretically ideal conditions that they are trained with an infinitely large dataset and receive an infinitely long context for prediction. We empirically verify that the effects of the two parameters, the training data size and context length, on the cross entropy consistently obey a power-law decay with a positive constant for two different state-of-the-art neural language models with different language datasets. Based on the verification, we obtained 1.12 bits per character for English by extrapolating the two parameters to infinity. This result suggests that the upper bound of the entropy rate of natural language is potentially smaller than the previously reported values.

## 1. Introduction

In recent years, a new type of computational model for natural language has emerged, the neural language model. The aggregation of technological advances in deep learning [1,2,3] has led to a series of substantial improvements to neural language models [4,5,6,7,8,9], and these models now significantly surpass the performance of *n*-gram language models in both character- and word-level prediction. Han et al. [10] further suggested that the performance of neural language models is potentially below currently reported values. They use neural networks with millions of parameters to best predict the next character from a context. The performance is quantified by the cross entropy, a measure of prediction accuracy.

Language modeling is a central task in natural language processing, and so language models have been developed and investigated for engineering purposes [11,12]. The majority of studies on neural language models has focused on decreasing the prediction error of the models in a fixed setting. We are naturally interested, however, in the case of situating models in extreme conditions such that an infinite amount of computational resources is available. Such a model’s prediction accuracy would be best if it was trained with an infinitely large dataset and made predictions by using an infinitely long context. The resulting value would, in fact, be a bound of the entropy rate.

The entropy rate of natural language is the average amount of information of one character in an infinite length of text, which characterizes the complexity of natural language. The entropy rate has been used to quantify the randomness of a process in many fields [13] and compare worldwide languages [14] specifically in computational linguistics. Because the true probability distributions of natural language are inaccessible, there have been various proposals for experimentally estimating the entropy rate. These approaches relied on the predictive power of humans [15,16] or computational models such as *n*-gram language models and compression algorithms [17,18,19]. Shannon [15] conducted a cognitive experiment in which a subject was asked to predict the next character from a previous context. He reported that the upper bound of the entropy rate of English is approximately 1.3 bits per character (bpc). Brown et al. [17] collected over 500 million words from text and constructed a word-level tri-gram language model to estimate the entropy rate of English characters. They reported an estimated upper bound of 1.75 bpc for English. They obtained this upper bound in a specific setting, i.e., with a fixed dataset size and context length.

In contrast to those studies, some studies have tried to estimate by extrapolating parameters to infinity. Hilberg [20], on the basis of the experimental results of Shannon [15], argued that the estimated value of the entropy rate would reach zero if a person received a text of infinite length to predict. While the majority of the studies [15,16,17,18,19] on this topic disagrees with this argument, it has motivated theoretical analyses [21,22,23,24] on estimating the entropy rate. Schümann and Grassberger [18] and Takahira et al. [19] sampled the encoding rate of a compression algorithm for different dataset sizes to estimate the encoding rate for an infinitely large dataset by extrapolation. Takahira et al. [19] observed that the accuracy of prediction follows relatively simple functions such as a power-law decay with a positive constant.

In this paper, we situate modern character-level neural language models in the problem of entropy rate estimation. We first study the effects of the model parameters, namely, the context length, training data size, and parameter size. Consistent with [19,25,26], we observe that the effects of the context length and training data size on the cross entropy consistently obey a power-law decay with a positive constant for two different neural language models with two different language datasets. On the basis of this observation, we extrapolate the parameters to infinity to obtain an estimate of the minimal cross entropy, which is a new bound of the entropy rate. We finally obtain an estimated entropy rate value of 1.12 bpc for English characters. This result suggests that the entropy rate of natural language is smaller than the previously reported values [15,16,17,19].

## 2. Entropy Rate Estimation of Natural Language

In this section, we introduce the terminology and concepts used in estimating the entropy rate of natural language with a language model.

**Definition** **1** (Shannon entropy)**.** *Let X be a stationary and ergodic stochastic process ${\left\{X_{t}\right\}}_{t=1}^{\infty }$, where each element takes a finite alphabet X and let $P(x_{1}^{m})$ be a joint probability distribution $P(X_{1}=x_{1},\ldots ,X_{m}=x_{m})$. The Shannon entropy of a stochastic process $H(X_{1}^{m})$ is defined as*

(1) $$H\left(X_{1}^{m}\right)=-\sum_{x_{1}^{m}\in X_{1}^{m}}P\left(x_{1}^{m}\right)\log P(x_{1}^{m}).$$

**Definition** **2** (Entropy rate)**.** *The entropy rate h of a stochastic process X is defined as*

(2) $$h=\lim_{m\to \infty }\frac{1}{m}H\left(X_{1}^{m}\right).$$

The entropy rate *h* is the averaged amount of information of an element of an infinite-length sequence.

The Shannon–McMillan–Breiman theorem states that almost surely

(3) $$h=\lim_{m\to \infty }-\frac{1}{m}\sum_{x_{1}^{m}\in X_{1}^{m}}\log P(x_{1}^{m}).$$

The entropy rate *h* is therefore equivalent to the average negative log-likelihood of one sample with a size of m→∞, $X_{1}^{m}$.

**Definition** **3** (Cross entropy and Kullback–Leibler divergence)**.** *The cross entropy L(P,Q) and Kullback–Leibler divergence KL(P∥Q) between the probability distribution P of stochastic process X and probability distribution of model Q are defined as follows.*

(4) $$L\left(P,Q\right)=-P\log Q=H\left(X_{1}^{m}\right)+KL\left(P∥Q\right)$$

(5) $$KL\left(P∥Q\right)=\sum_{x_{1}^{m}\in X_{1}^{m}}P\left(x_{1}^{m}\right)\frac{\log P(x_{1}^{m})}{\log Q(x_{1}^{m})}$$

One important property of the Kullback–Leibler divergence is Gibbs’ inequality, which states that

$$\begin{array}{cc} KL(P∥Q)\geq 0 & \\ KL(P∥Q)=0 & \mathrm{if}\;\mathrm{and}\;\mathrm{only}\;\mathrm{if}\;P=Q. \end{array}$$

From this property, the cross entropy is always greater than or equal to the entropy of a stochastic process *X*, and it is equal if and only if the two probability distributions, *P* and *Q*, are equivalent. Therefore, the cross entropy L(P,Q) is the upper bound of H(X). Similarly to Equation (3), we obtain Equation (6).

(6) $$L\left(P,Q\right)=\lim_{m\to \infty }-\frac{1}{m}\log Q(x_{1}^{m}).$$

We therefore obtain an upper bound of the entropy rate from the probability distribution of the model, *Q*.

**Definition** **4** (Language model)**.** *A language model with context length n outputs a conditional probability distribution Q:*

(7) $$Q(x_{i+1}|x_{i-n+1}^{i})$$

A language model with context length *n* computes the probability of the cross entropy as

(8) $$Q\left(x_{1}^{m}\right)=Q\left(x_{1},x_{2},\ldots ,x_{n-1}\right)\prod_{i=n}^{m}Q\left(x_{i}|x_{i-n+1},x_{i-n+2},\ldots ,x_{i-1}\right).$$

We still cannot compute L(P,Q) exactly, as thfe definition requires an infinitely long test dataset with m→∞. There are three parameters —the training data size *k*, context length *n*, and test data size *m*— for which we should specify infinite size, but this approach would not be practically computable. If a language model learned from a longer context length and training data size, however, it would better approximate the stochastic process of natural language, performing best when trained with an infinite context length and training data size. In Section 4 and Section 6, we discuss the effects of these parameters on estimating the upper bound of the entropy rate *h*.

## 3. Model

In this section, we introduce two neural language models and an *n*-gram language model, which are used in the experiment to estimate the entropy rate.

### 3.1. Neural Language Model

Neural language models are parameterized by neural networks. These models receive a string of characters of length *n* and output a probability distribution for the next character. They are optimized by minimizing the cross entropy. In this paper, we use two state-of-the-art neural language models: the Recurrent Highway Network (RHN) [5] for English characters, and Averaged Stochastic Gradient Descent Weight-Dropped LSTM with Mixture of Softmax (AWD-LSTM-MoS) [7,8], for Chinese characters. We chose these because they are the best-performing neural language models. The parameter sizes of the models are 46 million for RHN and 35 million for AWD-LSTM-MoS. In Section 5, we test the effect of the parameter size and confirm that a scale of tens of millions is sufficiently large for these models to achieve good performance. It requires more than a few months for one of the models to reach convergence with the datasets that we use. These neural language models use embedding representation and enriched architectures of recurrent neural networks (RNNs) and require proper training strategies to achieve state-of-the-art performance. We provide detailed explanations of the components and experimental setups of the neural language models in Appendix A.

### 3.2. *n*-Gram Language Model

To highlight the performance of the neural language models, we compare their results with those of *n*-gram language models, which are (n−1)-ordered Markov models. An *n*-gram model is often referred to as a count-based approach: the probability distribution is determined by the number of appearances in a training dataset.

In the experiment, we used a smoothing technique to enable longer *n*-grams and deal with data sparsity. Here, data sparsity refers to the fact that a large number of long *n*-grams in the test dataset never appeared in the training dataset. The smoothing technique calculates the weighted average of the probabilities of *n*-gram language models with different context lengths. The resulting model is a variant of the Katz backoff model [27]:

$$P_{n}\left(x_{i+1}|x_{i-n+1}^{i}\right)=\left\{\begin{array}{cc} l_{n}\frac{c(x_{i+1}|x_{i-n+1}^{i})}{c(x_{i-n+1}^{i})} & c(x_{i+1}|x_{i-n+1}^{i})>0\\ P_{n-1}\left(x_{i+1}|x_{i-n+2}\right) & c(x_{i+1}|x_{i-n+1}^{i})=0 \end{array}\right.$$

Here, The context $x_{i-n+1}^{i}$ is the elements of sequence *x* between the (i−n+1)th and *i*th elements, c(x|∗) is the count of *x* under condition ∗, and $l_{i}$ is the weight term for an *n*-gram that satisfies the normalization condition $\sum_{i=1}^{n}l_{i}=1$. This term is obtained by counting the number of times that $c(x_{i+1}|x_{i-n+1}^{i})>0$ is satisfied in a validation dataset separated from both the training and test datasets. The weight term $l_{i}$ allows the *n*-gram language model to balance the use of short and long contexts to best predict the next character. We do not use other smoothing techniques such as Kneser–Ney smoothing [28], because they are specialized for word-level modeling.

### 3.3. Dataset

We used two substantially large datasets: the one billion (OB) dataset [29] and the Central News Agency (CNA) corpus. The OB dataset is a collection of crawled news text in English. The CNA dataset is a similar collection of news text written in Chinese. The statistics of the datasets are listed in Table 1. These datasets are of the largest scale available to the best of our knowledge.

## 4. Estimating Entropy Rate Through Extrapolation

We are interested in the entropy rate of natural language, but we can only practically obtain an upper bound, the cross entropy L(P,Q), as mentioned in Section 2. Empirical studies on neural language models [25,26] have investigated the effects of experimental parameters on the cross entropy of models. Hestness et al. [25] investigated how the training data size affects the cross entropy of a neural language model. Likewise, Khandelwal et al. [26] investigated the effect of context length on the cross entropy of a model. These works demonstrate that the cross entropy of neural language models monotonically decreases as the context length and training data size increase.

The functional form of these effects has been studied empirically. In the context of entropy rate estimation, Hilberg [20] originally introduced $f_{0}$, a power-law function for Shannon’s figure in [15] that plots the entropy obtained from a cognitive experiment for different context lengths *n*. Hilberg claimed that $f_{0}$ with β≈0.5 fit the plot well and hypothesized that the entropy rate of English characters is equal to zero. He defined $f_{0}$ as

(9) $$f_{0}\left(x;A,\beta \right)=Ax^{\beta -1}$$

The power-law function $f_{0}$ is readily generalized to $f_{1}$:

(10) $$f_{1}\left(x;A,\beta ,h\right)=Ax^{\beta -1}+h.$$

The modified Hilberg function $f_{1}$ characterizes the cross entropy of a language model as a power-law decay with a positive constant. If h=0, then it is equivalent to $f_{0}$.

Hestness et al. [25] conducted empirical studies on the relationship between the training data size and a model’s performance in terms of three regions, called the “small-data region,” “power-law region,” and “irreducible-error region.” In the small-data region, neural network models only behave as random predictors. In the power-law region, the generalization error of a model decreases according to a power law. In the irreducible-error region, a model cannot reduce its error any more with a larger data size. Except for the small data region, this characterization is quite similar to the modified Hilberg function $f_{1}$ because the terms $x^{\beta -1}$ and *h* stand for power-law decay and irreducible error, respectively. These empirical observations could potentially be explained by statistical learning theory [30], although the settings of existing works [31] are largely different from language modeling.

With respect to these previous findings, in the rest of this paper, we first validate whether the effects of context length and training data size on language models really follow the modified Hilberg function $f_{1}$ and the observations by Hestness et al. [25]. Then, we extrapolate the experimental results to estimate the cross entropies of language models at an infinite context length and infinite training data size. Possible functional forms were discussed in [19], which found that $f_{0}$ and $f_{1}$ are the most proper functions. This paper therefore focuses on $f_{0}$ and $f_{1}$ and excludes other fitting functions proposed in [18,19]. The parameters of $f_{0}$ and $f_{1}$ are obtained by minimizing the mean square-root error ϵ:

(11) $$\epsilon =\frac{1}{s}\sqrt{\sum_{i=1}^{s}{\left(f\left(x_{i}\right)-y_{i}\right)}^{2}}$$

where $x_{i}$ is the *i*th data point of a training data size or context length, $f(x_{i})$ is the cross entropy predicted by function *f* at $x_{i}$, and $y_{i}$ is the cross entropy of a model with the condition of $x_{i}$.

## 5. Effect of Parameters

### 5.1. Effect of Context Length *n*

**Figure 1:**
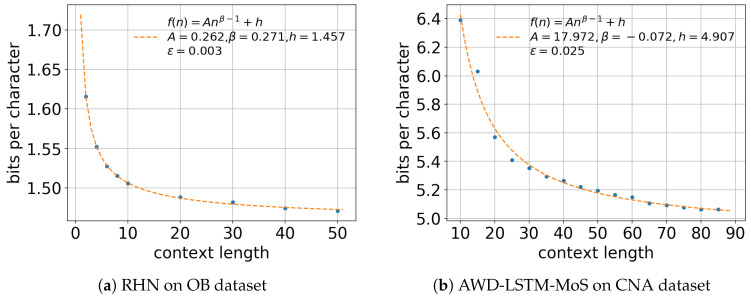
Effect of context length on the cross entropy of (**a**) RHN trained on 16,777,216 characters of the OB dataset and (**b**) AWD-LSTM-MoS trained on 8,388,608 characters of the CNA dataset. Both of these neural language models improved their prediction accuracy with longer context and were well characterized by the modified Hilberg function $f_{1}$.

We first investigated the effect of context length on the cross entropy of the language models. We trained the models with different context lengths and sampled the cross entropy for each model. Figure 1 shows the relationship between the context length and cross entropy for RHN on the OB dataset and AWD-LSTM-MoS on the CNA dataset. For RHN on the OB dataset, we trained with different context lengths (2,4,6,8,10,20,30,40,50) and 16,777,216 characters of the OB dataset. For AWD-LSTM-MoS, we conducted the same experiment with different context lengths (5,10,…,85) and 8,388,608 characters of the CNA dataset. We had shorter context lengths for RHN because it could not run on a single GPU with long context lengths over 50. These neural language models improved their cross entropy by using context lengths on the order of several tens.

**Figure 2:**
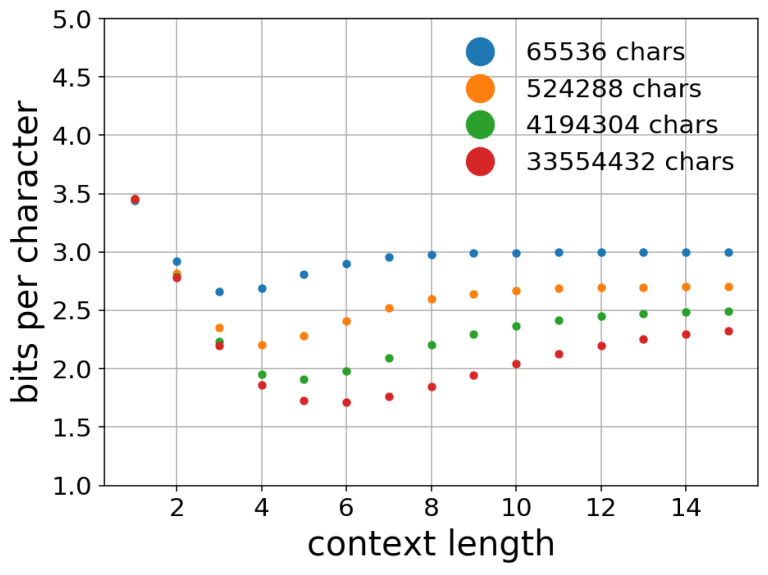
Effect of context length on the cross entropy of *n*-gram (1–15-gram) models with different training data sizes of 65,536, 524,288, 4,194,304, and 33,554,432 characters. A larger training data size allowed the *n*-gram language models to improve their prediction accuracy w.r.t. the context length. Even with such large training datasets, however, the best performing *n*-gram model had n≤10.

In contrast, the *n*-gram language models did not benefit from longer contexts to improve their prediction accuracy. Figure 2 shows a scatter plot of the context length and cross entropy with different training data sizes. With larger training data, the best performing value of “*n*” increased: with 65,536 characters, n=3 performed best, whereas with 33,554,432 characters, n=6 performed the best. Even with the largest dataset, however, the *n*-gram models did not benefit from contexts longer than n≈10 to improve their cross entropy. This difference characterizes the advantage of neural language models over *n*-gram language models.

### 5.2. Effect of Training Data Size k

**Figure 3:**
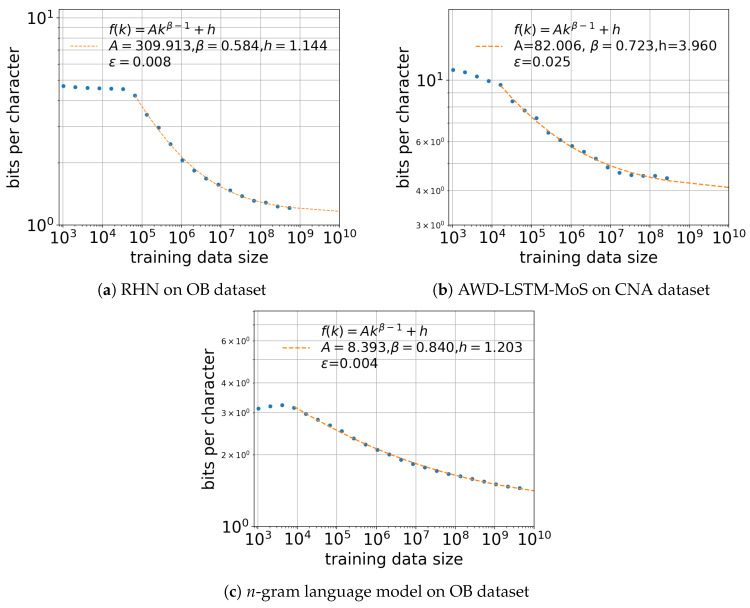
Cross entropy of (**a**) RHN on OB dataset, (**b**) AWD-LSTM-MoS on CNA dataset, and (**c**) *n*-gram language models on OB dataset with different training data sizes. The fitting function $f_{1}$ was applied to the data in the regions of k≥65,536 for RHN, k≥16,384 for AWD-LSTM-MoS, and k≥8192 for the *n*-gram language models. The effect of the training data size was well characterized by $f_{1}$, as with the context length in Figure 1.

We next conducted an experiment to examine the relationship between the training data size and the cross entropy of the neural and *n*-gram language models. Figure 3 shows the relationships between the dataset size and cross entropy for the various models with different training data sizes. For the neural language models, we recorded the cross entropy with different dataset sizes $\left(2^{10},2^{11},\ldots \right)$. The context lengths were fixed to sufficiently large lengths of 50 for RHN on the OB dataset and 70 for AWD-LSTM-MoS on the CNA dataset. For the *n*-gram language models, we obtained the cross entropy for n=1,…,8 and the different training data sizes and selected the lowest cross entropy for each size.

For the results shown in Figure 3, because the models each exhibited a small-data region in which the cross entropy did not decrease or decay slowly, we defined *drop point* values at which the small-data region ended and the power-law region began. These values were determined heuristically. We then computed the parameters of $f_{1}$ from the sampled data points after the *drop points*.

Our results confirm that the modified Hilberg function well characterizes the effect of the training data size on the cross entropy. RHN only made random predictions up to a training data size of $10^{5}$ characters. The *n*-gram language models also had a small-data region up to $10^{4}$ characters. These models then improved their cross entropies monotonically with increased training data size. This relationship was consistent for the different models and languages, as seen in Figure 3.

One notable difference was the behavior in the small-data region. While RHN on the OB dataset showed a plateau, AWD-LSTM-MoS on the CNA dataset had a slower but monotonic decay even in this region. One reason for this difference is the gap in the number of unique characters in English (139) and Chinese (9171). In the case of Chinese, if the training data size is significantly small, the model will be trained to output a small portion of unique characters. As the size increases, the model is more likely to process a larger ratio of unique characters, which leads to improved prediction accuracy. Therefore, there was a slight slope in the small-data region for AWD-LSTM-MoS on the CNA dataset.

### 5.3. Effect of Test Data Size *m*

By definition, the cross entropy L(P,Q) requires an infinitely long text, but we had only texts of finite length to obtain cross entropy values. In general, it is difficult to construct confidence bounds for the obtained values from neural networks. Because of this problem, it is uncertain whether the obtained values are reliable estimates. Therefore, we investigated the effect of the test data size *m* on the values obtained.

**Figure 4:**
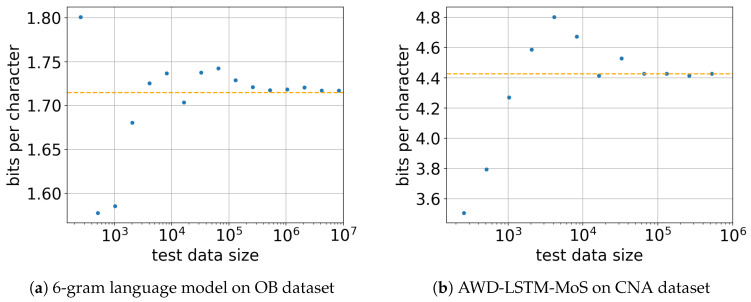
Effect of test data size on the cross entropy values. The models were trained with 33,554,432 characters of the corresponding datasets and evaluated with different test data sizes. The orange lines indicate the cross entropies obtained with the test datasets of maximum length. For both models (**a**,**b**), the values seemed to converge when the test data size was sufficiently large ($m>10^{5}$).

To cover different datasets and models, we examined the effect of the test data size on a 6-gram language model on the OB dataset and AWD-LSTM-MoS on the CNA dataset. The models were trained with 33,554,432 characters of the corresponding datasets. Figure 4 shows the values sampled at different test data sizes $\left(2^{10},2^{11},\ldots \right)$. The orange lines in the figures represent the values obtained with the largest test data sizes. When the test data size was too small, up to $m\approx 10^{5}$, the obtained values fluctuated for both the 6-gram language model and AWD-LSTM-MoS, making the results unreliable. The values then seemed to converge when the test data size was sufficiently large, over $10^{5}$ characters. Although this empirical analysis does not guarantee convergence, in practice, we can fairly compute the cross entropy L(P,Q) with $m>10^{5}$ characters of text.

### 5.4. Effect of Parameter Size

**Figure 5:**
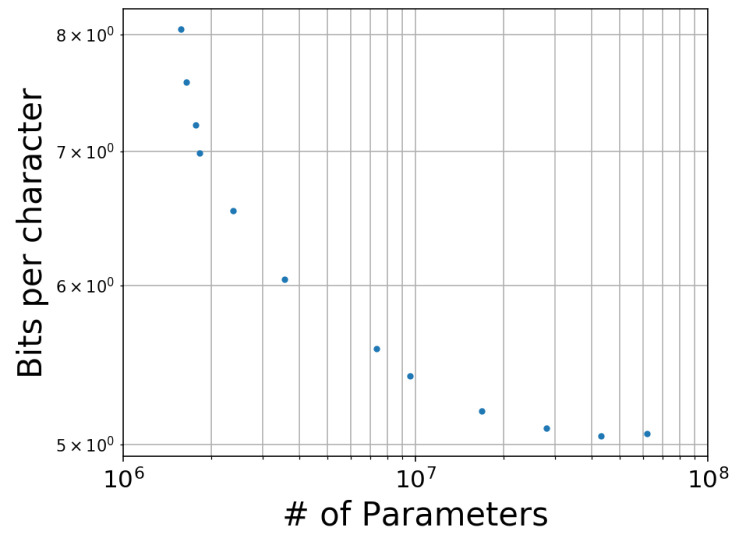
Effect of parameter size on the cross entropy for AWD-LSTM-MoS with the CNA dataset. The parameter size of AWD-LSTM-MoS was controlled by changing the numbers of dimensions of the LSTM layers. Even the minimum parameter size exceeded $10^{6}$, because the vocabulary size of Chinese made the softmax layer of AWD-LSTM-MoS large. The cross entropy reached a plateau at a scale of $10^{7}$ parameters.

The parameter size of neural language models (and of deep learning models in general) has a strong effect on their performance. We empirically analyzed this effect for AWD-LSTM-MoS with 4,194,304 characters of the CNA dataset. We varied the number of parameters by changing the numbers of dimensions of the LSTM layers and trained the models until convergence. Figure 5 shows a scatter plot of the number of parameters and the cross entropy. Similarly to the observations for the context length and training data size, the performance monotonically increased up to approximately $10^{8}$, but then the values seemed to converge around that parameter size. Therefore, we used a model with a fixed number of parameters for the experiment. Note that we excluded RHN here because that model with a larger parameter size could not run on a single GPU.

## 6. Entropy Rate Estimated with Neural Language Models

From the above observations, we considered three approaches for estimating the entropy rate with the neural language models. The first approach simply uses the best, smallest cross entropy recorded by a model. This is the standard method for measuring the performance of language models, because the cross entropy is the upper bound of the entropy rate. Table 2 summarizes the smallest cross entropies recorded by the three models, as well as by the PPM-d algorithm [19]. We obtained 1.21 bpc for English and 4.43 bpc for Chinese from the respective RHN and AWD-LSTM-MoS models. These values were significantly smaller than those for the *n*-gram language models and the PPM-d algorithm, especially for the CNA dataset in Chinese.

The second approach uses extrapolation of the training data size with fixed context lengths and $f_{1}$. We prioritized the training data size over the context length, because context lengths of n≥50 for RHN and n≥70 for AWD-LSTM-MoS had a small impact on the cross entropy, as seen in Figure 1. As summarized in Table 3, we obtained 1.14 bpc for the OB dataset (English) and 3.96 bpc for the CNA dataset (Chinese), which are smaller cross entropy values than with the first approach.

The third approach extends the modified Hilberg function $f_{1}$ to a bivariate function *g* to take both the context length and dataset size into account:

(12) $$g\left(x_{1},x_{2};A_{1},A_{2},\beta_{1},\beta_{2},h\right)=A_{1}x_{1}^{\beta_{1}}+A_{2}x_{2}^{\beta_{2}}+h$$

where the parameter $x_{1}$ is the training data size, and $x_{2}$ is the context length. This extension from $f_{1}$ to *g* allows us to estimate the entropy rate *h* from the power-law decay of the cross entropy with respect to both the context length and dataset size. This was a natural extension of the second approach, as we found that $f_{1}$ well described the cross entropy of the models with respect to those two parameters. Because of the substantial increase in the number of sample points and the heavy computational cost for training the models, we focused on RHN with the OB dataset for this approach. For this bivariate regression, we sampled the cross entropy of RHN for all points at various context lengths (2,4,6,8,10,20,30,40) and training data sizes ($2^{20},\ldots ,2^{25}$), and at a context length of 50 with various dataset sizes $\left(2^{20},\ldots ,2^{29}\right)$.

**Figure 6:**
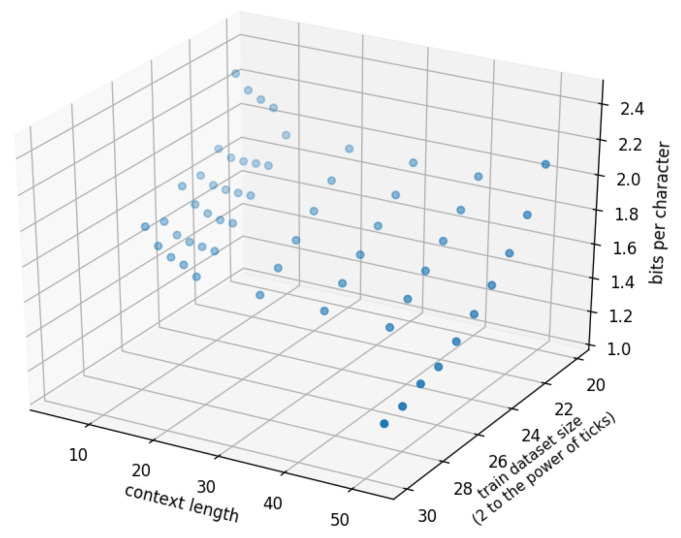
Cross entropy of RHN (z-axis) with different context lengths (x-axis) and training data sizes (y-axis) on the OB dataset. The data was sampled at various context lengths (2,4,6,8,10,20,30,40) and training data sizes ($2^{20},\ldots ,2^{25}$), and at a context length of 50 with various dataset sizes $\left(2^{20},\ldots ,2^{29}\right)$. The data was fitted by function *g* with the parameters in Table 4.

Figure 6 shows a 3D scatter plot of the cross entropy of RHN with different context lengths and training data sizes on the OB dataset. The parameters of *g* are listed in Table 4. With this setting, we obtained 1.12 bpc for the OB dataset in English. This value is smaller than those obtained in previous reports and with the above two estimation approaches. Note, however, that the parameter fitting is sensitive to deficits of data or changes in the range of samples, as compared with the previous two approaches.

## 7. Conclusions

We explored estimation of the entropy rate of natural language with neural language models. We investigated the effects of four key parameters: the context length, training data size, test data size and parameter size. With respect to our findings on entropy rate estimation and empirical evaluation, we verified that the cross entropy of the neural language models decreased with a longer context length and larger training data size, and the decreases were characterized with the modified Hilberg function $f_{1}$. These observations characterize how a machine learning model improves its performance as it receives more data for training. We then empirically explored the asymptotic behavior with a test data size of $m>10^{5}$ characters. We also tested the effect of varying the number of parameters. Through regression with the results of these investigations, we finally obtained entropy rate upper bounds of 1.12 bpc for English and 3.96 bpc for Chinese. These values are smaller than the previously reported values obtained with other computational models. 

## Figures and Tables

**Table 1:** Statistics of the datasets used in the experiment.

Dataset	Character Tokens	Word Tokens	Vocabulary	Unique Characters	Test Character Tokens
One Billion (English)	4,091,497,941	768,648,885	81,198	139	37,811,222
Central News Agency (Chinese)	743,444,075	415,721,153	1,694,159	9171	493,376

**Table 2:** Smallest cross entropy values obtained by the neural language models, *n*-gram language models, and PPM-d algorithm. The values were obtained from the models trained with the largest training data size and longest context length in the experiment. The neural language models consistently outperformed the *n*-gram language models and the PPM-d algorithm.

Dataset	Model	Smallest Cross-Entropy
OB	RHN	**1.210**
OB	*n*-gram model	1.451
OB	PPM-d	1.442
CNA	AWD-LSTM-MoS	**4.429**
CNA	*n*-gram model	4.678
CNA	PPM-d	4.753

Note: The smallest cross entropies for the two datasets are in bold.

**Table 3:** Estimated parameters and error of the fitting function $f_{1}$ for the language models (including the PPM-d algorithm [19]) with different training data sizes. This fitting corresponds to the second approach described in Section 6. The drop point indicates the minimum training data size for fitting with the modified Hilberg function $f_{1}$.

Dataset	Model	$f_{1}$				Drop Point
		*A*	β	*h*	ϵ	
OB	RHN	358.997	0.570	**1.144**	0.008	$2^{16}$
OB	*n*-gram model	8.393	0.840	1.210	0.004	$2^{13}$
OB	PPM-d	12.551	0.791	1.302	0.015	-
CNA	AWD-LSTM-MoS	82.006	0.723	**3.960**	0.025	$2^{14}$
CNA	*n*-gram model	388.338	0.647	4.409	0.018	$2^{22}$
CNA	PPM-d	35.602	0.766	4.295	0.078	-

Note: The smallest cross entropies for the OB and CNA datasets are in bold.

**Table 4:** Estimated parameters and error of fitting function *g*. This fitting corresponds to the third approach described in Section 6.

Dataset	Model	*g*					
		$A_{1}$	$\beta_{1}$	$A_{2}$	$\beta_{2}$	*h*	ϵ
1B	RHN	89.609	0.661	0.324	0.294	**1.121**	0.006

Note: The estimated value of the entropy rate *h* with the bivariate regression is marked in bold.

## Appendix A. Neural Language Models

In this Appendix, we explain the general structures of modern neural language models and the specifications of Recurrent Highway Networks (RHNs) [5] and the AWD-LSTM-MoS model [8], which we used in the experiment. Modern neural language models rely on three components: an embedding representation, enriched architectures of recurrent neural networks (RNNs), and training strategies. In the following equations, bold capital letters such as W and U are matrices, bold lowercase letters such as h and b are one-dimensional vectors, and normal letters are real values.

### Appendix A.1. Embedding Representation

In the scheme of machine learning, discrete symbols are represented by one-hot vectors. In the case of the character language models that we used, “a” is represented with (1,0,0,…,0), “A” is represented with (0,1,0,…,0), and so on. Therefore, “a” and “A” are represented as independent forms. This embedding representation allows us to describe the similarity and difference between “a” and “A” quantitatively. An embedding layer projects the discrete one-hot vector representation to a $d_{\mathrm{emb}}$-dimensional continuous space. The vector of the corresponding symbol relatively describes the similarity and difference between the symbols. Embedding representation is a key to many successful applications of deep learning in natural language processing. In language modeling, the vectors of an embedding layer are initialized randomly and updated by a stochastic gradient algorithm together with the other layers of the neural language model.

### Appendix A.2. Enriched Architectures of Recurrent Neural Networks

The RNN is a suitable architecture for neural networks with sequential data. An RNN takes the elements at time step *t*, $x_{t}$ (the embedding representation of a character), and a hidden vector $h_{t-1}$ as inputs to generate an output $h_{t}$:

(A1) $$h_{t}=f\left(Ux_{t}+Wh_{t-1}+b\right)$$

where *f* is a non-linear activation function, U and W are weight matrices, and b is a constant term. Plain RNNs, however, suffer from difficulty in training. Modern studies with RNNs have used enriched versions of the networks such as long short-term memory (LSTM). They have been widely applied to many tasks in, including language modeling. In contrast to plain RNNs, an LSTM has a cell $c_{t}$ and gating mechanisms. The computation process of LSTM is written as follows.

(A2) $$\begin{array}{ccc} i_{t} & = & \sigma (U_{i}x_{t}+W_{i}h_{t-1}+b_{i}) \end{array}$$

(A3) $$\begin{array}{ccc} f_{t} & = & \sigma (U_{f}x_{t}+W_{f}h_{t-1}+b_{f}) \end{array}$$

(A4) $$\begin{array}{ccc} o_{t} & = & \sigma (U_{o}x_{t}+W_{o}h_{t-1}+b_{o}) \end{array}$$

(A5) $$\begin{array}{ccc} {\tilde{c}}_{t} & = & \tanh(U_{\tilde{c}}x_{t}+W_{\tilde{c}}h_{t-1}+b_{\tilde{c}}) \end{array}$$

(A6) $$\begin{array}{ccc} c_{t} & = & \sigma (f_{t}*c_{t-1}+i_{t}*{\tilde{c}}_{t}) \end{array}$$

(A7) $$\begin{array}{ccc} h_{t} & = & \tanh\left(c_{t}\right)*o_{t}. \end{array}$$

where ∗ denotes element-wise multiplication, σ is a sigmoid function, and $i_{t}$, $f_{t}$, and $o_{t}$ correspond to an input gate, forget gate, and output gate, respectively. In contrast to plain RNNs, as described in Equations (A5) and (A6), an LSTM is allowed to balance the use of contextual information from the previous time step, $c_{t-1}$, and the standard computation in an RNN, ${\tilde{c}}_{t}$. This is called a gating mechanism.

### Appendix A.3. Training Strategy

Neural language models require a proper training strategy to achieve state-of-the-art performance. Here, we introduce “dropout” and a “scheduled learning rate” as techniques of training, because they are the most impactful factors for improving the prediction performance of neural language models [9]. Dropout [3] is the most popular and effective regularization technique for neural networks. The idea is to randomly drop units from a network. Dropout prevents neural networks from overfitting and contributes to improving the generalization performance. In the case of RNNs, a plausibly modified variant called variational dropout [32] is used. A scheduled learning rate is another key to training neural networks effectively. In many successful deep learning models, the learning rate is set to a large value at the early stage of training and scheduled to decay as training progresses.

### Appendix A.4. Recurrent Highway Networks

We used the official RHN implementation coded in *tensorflow 0.12* [33] and available at https://github.com/julian121266/RecurrentHighwayNetworks. The RHN is an enriched RNN architecture inspired by highway networks [34]. It introduces *deep* computation within one time step of an RNN and has *L* layers to compute a hidden vector at the next time step. Following the notation in the original paper, the RHN computation is defined as

(A8) $$\begin{array}{ccc} h_{l,t} & = & \tanh(U_{h}x_{t}1_{\left\{l=L\right\}}+W_{h_{l}}s_{l-1,t}+b_{h_{l}}) \end{array}$$

(A9) $$\begin{array}{ccc} t_{l,t} & = & \sigma (U_{t}x_{t}1_{\left\{l=L\right\}}+W_{t_{l}}s_{l-1,t}+b_{t_{l}}) \end{array}$$

(A10) $$\begin{array}{ccc} c_{l,t} & = & \sigma (U_{c}x_{t}1_{\left\{l=L\right\}}+W_{c_{l}}s_{l-1,t}+b_{c_{l}}) \end{array}$$

(A11) $$\begin{array}{ccc} s_{l,t} & = & h_{l,t}*t_{l,t}+s_{l-1,t}*c_{l,t} \end{array}$$

where $1_{\left\{\right\}}$ is an indicator function. The first RHN layer takes $x_{t}$ and $s_{L,t-1}$ as inputs to produce its output. The following layers take $s_{l,t}$ as an input to output $s_{l+1,t}$. The RHN layers finally compute $s_{L,t}$, which is regarded as the hidden vector in LSTM, $h_{t}$. Here, $h_{l,t}$ corresponds to the computation in a standard RNN, and $t_{l,t}$ and $c_{l,t}$ function as gating mechanisms to control the use of $h_{l,t}$ and $s_{l-1,t}$ in computing $s_{l,t}$. Therefore, the RHN is regarded as an extension of LSTM.

In the experiment, the number of layers was set to 10. The number of RHN units was set to 1500, and the total number of parameters reached 46 million. Stochastic gradient descent with momentum [35] was used as the optimization algorithm. The initial learning rate was set to 0.2, and a learning-rate decay of 1.03 was applied for every five epochs. A weight decay of $10^{-7}$ was applied for regularizing the parameters. Variational dropout was also applied, as well as another technique called gradient clipping, in which the norm of a gradient was rescaled to 10 when it exceeded 10.

### Appendix A.5. AWD-LSTM-MoS

The AWD-LSTM-MoS model is an extension of the AWD-LSTM implemented in *PyTorch 0.4* [36]. The official implementation is available at https://github.com/zihangdai/mos. In contrast to an RHN, AWD-LSTM keeps the model ingredients simple (embedding layer + LSTM layer + softmax layer) and focuses on the training strategy to improve the model performance. AWD-LSTM-MoS makes a change to the softmax layer. The standard softmax layer receives $h_{t}$ to output the probability of the next character as

(A12) $$P\left(y=w|h_{t}\right)=\frac{\exp(h_{t}^{T}w_{w})}{\sum_{w^{\prime }}\exp(h_{t}^{T}w_{w^{\prime }})}.$$

The numerator represents the likelihood that y=w, and the denominator is a normalization constant to satisfy $\sum_{w^{\prime }}P\left(y=w^{\prime }|h_{t}\right)=1$. A softmax layer is the standard choice for the output layer of a classification model. In a previous paper, we proposed a notion called mixture of softmax (MoS). As the name suggests, an MoS layer computes the probability of the next character from a mixture of *K* different softmax computations as follows.

(A13) $$\begin{array}{ccc} h_{k,t} & = & \tanh(W_{k}h_{t}) \end{array}$$

(A14) $$\begin{array}{ccc} \pi_{k} & = & \frac{\exp(h_{t}^{T}w_{\pi ,k})}{\sum_{k^{\prime }=1}^{K}\exp(h_{t}^{T}w_{\pi ,k^{\prime }})} \end{array}$$

(A15) $$\begin{array}{ccc} P(y=w|h_{t}) & = & \sum_{k=1}^{K}\pi_{k}\frac{\exp(h_{c,k}^{⊤}w_{w})}{\sum_{w^{\prime }}\exp\left(h_{c,k}^{⊤}w_{w^{\prime }}\right)} \end{array}$$

The authors argued that MoS contributes to the expressibility of a softmax layer. The experimental result demonstrated that it leads to an improved performance in a language modeling task.

In the experiment, K=15 was chosen. For the rest of the hyperparameters, we set the dimension of the embedding layer $d_{\mathrm{emb}}$ to 300, and the three layers of the LSTM had 1150, 1150, and 650 dimensions. The total number of parameters reached 35 million. Gradient clipping was applied for gradients with norms larger than 0.25. Averaged stochastic gradient descent (ASGD) [37] with an initial learning rate of 15.0 was used to update the weight with a batch size of 15. Variational dropout was also applied to the layers of this model.
